# Management of children with danger signs in integrated community case management care in rural southwestern Uganda (2014–2018)

**DOI:** 10.1093/inthealth/ihad039

**Published:** 2023-06-07

**Authors:** Michael Matte, Moses Ntaro, Jessica Kenney, Palka Patel, Andrew Christopher Wesuta, Peter Chris Kawungezi, Shem Bwambale, David Ayebare, Stephen Baguma, Fred Bagenda, James S Miller, Geren Stone, Edgar Mugema Mulogo

**Affiliations:** Department of Community Health, Mbarara University of Science and Technology, PO Box 1410, Mbarara, Uganda; Department of Community Health, Mbarara University of Science and Technology, PO Box 1410, Mbarara, Uganda; Center for Global Health, Massachusetts General Hospital, 125 Nashua Street, Boston, MA 02114, USA; Indiana University School of Medicine, 340 West 10th Street Fairbanks Hall, Suite 6200 Indianapolis, IN 46202-3082, USA; Bugoye Community Health Collaboration, Bugoye Health Centre III, PO Box 149, Kasese, Uganda; Department of Community Health, Mbarara University of Science and Technology, PO Box 1410, Mbarara, Uganda; Bugoye Community Health Collaboration, Bugoye Health Centre III, PO Box 149, Kasese, Uganda; Department of Community Health, Mbarara University of Science and Technology, PO Box 1410, Mbarara, Uganda; Bugoye Community Health Collaboration, Bugoye Health Centre III, PO Box 149, Kasese, Uganda; Department of Community Health, Mbarara University of Science and Technology, PO Box 1410, Mbarara, Uganda; Center for Global Health, Massachusetts General Hospital, 125 Nashua Street, Boston, MA 02114, USA; Center for Global Health, Massachusetts General Hospital, 125 Nashua Street, Boston, MA 02114, USA; Department of Community Health, Mbarara University of Science and Technology, PO Box 1410, Mbarara, Uganda

**Keywords:** Bugoye Community Health Collaboration, Community Health Workers, Integrated Community Case Management, Uganda

## Abstract

**Background:**

In integrated community case management (iCCM) care, community health workers (CHWs) provide home-based management of fever, diarrhea and fast breathing for children aged <5 y. The iCCM protocol recommends that children with danger signs for severe illness are referred by CHWs to health facilities within their catchment area. This study examines the management of danger signs by CHWs implementing iCCM in a rural context.

**Methods:**

A retrospective observational study that examined clinical records for all patients with danger signs evaluated by CHWs from March 2014 to December 2018 was conducted.

**Results:**

In total, 229 children aged <5 y had been recorded as having a danger sign during 2014–2018. Of these children, 56% were males with a mean age of 25 (SD 16.9) mo, among whom 78% were referred by the CHWs as per the iCCM protocol. The age category of 12 to 35 mo had the highest numbers of prereferred and referred cases (54% and 46%, respectively).

**Conclusions:**

CHWs play a key role in early symptomatic detection, prereferral treatment and early referral of children aged <5 y. Danger signs among children aged <5 y, if left untreated, can result in death. A high proportion of the children with danger signs were referred as per the iCCM protocol. Continuous CHW training is emphasized to reduce the number of referral cases that are missed. More studies need to focus on children aged 12–35 mo and why they are the most referred category. Policymakers should occasionally revise iCCM guidelines to detail the types of danger signs and how CHWs can address these.

## Introduction

Malaria, diarrhea and pneumonia have remained prevalent among children aged <5 y. Globally, up to 45% of deaths among children aged <5 y are attributed to malaria, diarrhea and pneumonia. In sub-Saharan Africa, 41% of deaths come from these three preventable diseases, which have also contributed to childhood morbidity.^1–5^

In many parts of Africa, the majority of deaths in children aged <5 y occur in the community before a health facility is accessed.^2^ Uganda adopted home-based management of fever that was introduced in 2002 and operated through volunteer community-based distributors who offer free prepackaged chloroquine and sulfadoxine-pyrimethamine. Shortages in the then new first line therapy artemether-lumefantrine resulted in the program failing to proceed. In 2010, motivated by a malaria consortium, UNICEF and the WHO, a more supported approach, including training, supervision and availability of supplies, was slowly rolled out in parts of supportive counties.^6^ The integrated community case management (iCCM) strategy has since been observed largely as a success, not only in Uganda, but in many countries on the continent.^7–9^

iCCM care allows for quick and reachable management and treatment of children brought to community health workers (CHWs) with fever, diarrhea and fast breathing, particularly in rural settings, where patients and households face topographical barriers to accessing facility-based care.^10–12^ However, some children will still present to CHWs with signs of severe illness and require prompt referral to health facility-based care. The danger signs in iCCM include (but are not limited to) seizures, not being able to drink or breastfeed, severe vomiting, respiratory distress and an altered level of consciousness.^13,14^

CHWs have been found to be more accessible and more generally available according to numerous studies in comparison with some health facilities that permit the management of danger signs among patients seeking medical services.^15^ Community trust and confidence are built into many aspects of how CHWs are trained. In turn, continuous management of community cases by CHWs increases community knowledge of danger signs, encouraging CHW-based referral of complicated cases.^16^

In Uganda in particular, the mortality rates for children aged <5 y for malaria (13%), diarrhea (8%) and respiratory infections, including pneumonia (15%), are still significantly high, despite the efforts of the government of Uganda and partners.^4^ In Kasese District, Bugoye subcounty, iCCM has been implemented with fully trained CHWs since March 2013; this followed the Ugandan Ministry of Health’s establishment of national iCCM implementation guidelines in 2010, with CHWs providing iCCM care.^17^ Data were collected on a monthly basis by CHWs; however, there is limited information on appropriate management of danger signs for fever, diarrhea and fast breathing by CHWs.

Children who present to CHWs with danger signs need to be attended to urgently, given appropriate prereferral treatment in accordance with iCCM guidelines, then referred to care from professional medical workers to avoid morbidity or even mortality. If left unattended, children aged <5 y with danger signs may succumb to death. World Health Organization (WHO) Sustainable Development Goal 3 (Target 3.2) emphasizes ending preventable mortality in children aged <5 y. The current study examined the management of danger signs, prereferral practices and referral outcomes by CHWs in children aged <5 y in Bugoye subcounty. This study adds to the existing body of knowledge and informs policymakers at the Ministry of Health regarding the management of children with danger signs by CHWs in a rural context.

## Methods

### Study setting

Bugoye subcounty is located in the Kasese district of western Uganda. Its mountainous geography limits access to facility-based care for some residents. The subcounty has a population of approximately 46 124 residents and 7650 households distributed within 35 villages. The population of children aged <5 y is 9225 (20%).^18^ Many residents rely on subsistence farming and small-scale coffee farming.

The Bugoye Community Health Collaboration (BCHC) is a partnership between Bugoye Health Center, Mbarara University of Science and Technology and Massachusetts General Hospital. The BCHC initially established iCCM care in five villages (with 25 CHWs) in 2013 that was then expanded to include an additional eight villages. All CHWs are volunteers selected by their communities, who are trained for 1 wk in iCCM based on Uganda Ministry of Health guidelines. During the period covered by the current study, CHWs used paper-based iCCM registers to document patient visits and danger signs and submitted these registers each month to the BCHC.^19^

The iCCM protocol states that all children aged <5 y reporting to a CHW are diagnosed for fever, diarrhea and fast breathing, based on what the caregiver presents. The iCCM general danger signs of convulsions, vomiting up everything, not breastfeeding, not being able to drink/eat and being very sleepy/unconscious, necessitate immediate referral to a nearby health facility.^19,20^ In the current study, appropriate management occurs when the CHW identifies a danger sign and refers that case to the nearest health facility.

### Data collection

The iCCM registers include basic demographic information, presenting complaints and CHWs’ clinical assessments, as well as information on treatment and danger signs. Using these registers, the BCHC data team created a clinical database of all iCCM patient visits from April 2014 to December 2018 comprising 18 430 visits. Data were aggregated, entered into Epidata 3.1 software and stored in Research Electronic Data Capture, (Research electronic data capture (REDCap)—A metadata-driven methodology and workflow process for providing translational research informatics support). These data were cleaned and validated for inconsistencies and completeness. For this study, data for the 229 patients with danger signs identified by CHWs were then extracted from the overall database. The 229 records consist of all the records with danger signs. This dataset is available for any required review.

### Data analysis

Data were exported from Excel to STATA version 15 (Statacorp, College Station, TX, USA) for analysis to determine appropriate management based on the iCCM algorithm and subsequent descriptive analyses were also conducted.

## Results

### Demographic information and danger signs presented

Overall, 229 children aged <5 y presented with a danger sign and were therefore considered for analysis. Of the 229 children with a danger sign, 56% were male, with an approximately even distribution by age; the mean age was 25 (SD 16.9) mo. Children aged 12–35 mo (43%) were the most affected age category. Fever (43%) was the most common complaint presented, followed by diarrhea (12%), then cough/fast breathing (7%). Twenty-two children (10%) aged <5 y presented with danger signs of fever and cough and were managed by CHWs (Table 1).

**Table 1.** Background characteristics of children with danger signs

Measure	n (%)
Number of children aged <5 y seen by CHWs	18 430
Total encounters with danger signs	229
Male children aged <5 y	128 (56%)
Age categories*	
2–11 mo	44 (19%)
12–35 mo	99 (43%)
36–60 mo	82 (36%)
*Presenting complaints*	
Fever only	80 (35%)
Cough/fast breathing only	15 (7%)
Diarrhea only	28 (12%)
Multiple complaints (e.g. fever and cough/fast breathing)	41 (18%)
Other complaints (apart from fever, cough/fast breathing or diarrhea)	65 (28%)
*Children aged <5 y with multiple presenting complaints*	
Fever and cough/fast breathing	22 (10%)
Fever and diarrhea	10 (4%)
Cough/fast breathing and diarrhea	5 (2%)
Fever, cough/fast breathing and diarrhea	4 (2%)

*There were four patients whose ages were missing.

### Management of children with danger signs by CHWs

Among children aged <5 y presenting with danger signs, fever (54%) was the most common condition managed by CHWs for which prereferral treatment was provided. Seventy-eight patients (78%) who had fever were referred to a health facility by CHWs. Of the 28 diarrhea patients, 21 were appropriately managed by CHWs. Only 6 out of 15 fast breathing patients were appropriately managed by CHWs. Even among the 65 patients with other complains, 89% were referred appropriately by CHWs to the nearest health facility (Table 2).

**Table 2.** Appropriate management of children with fever, diarrhea and fast breathing danger signs

Measure	n (%)
*Children aged <5 y referred for facility-based care*	
Children aged <5 y with fever only (n=80)	62 (78%)
Children aged <5 y with cough/fast breathing only (n=15)	6 (40%)
Children aged <5 y with diarrhea only (n=28)	21 (75%)
Children aged <5 y with multiple complaints (n=41)	30 (73%)
Children aged <5 y with other complaints (n=65)	58 (89%)
All children aged <5 y with danger signs (n=229)	177 (77%)
*Appropriate prereferral treatment given to eligible children aged <5 y*	
Children aged <5 y with fever only (n=80)	43 (54%)
Children aged <5 y with cough/fast breathing only (n=15)	8 (53%)
Children aged <5 y with diarrhea only (n=28)	16 (57%)
Children aged <5 y with multiple complaints (n=41)	33 (80%)
All eligible children aged <5 y (n=164)	100 (61%)
*Children aged <5 y receiving appropriate prereferral treatment (if indicated) and referral to facility-based care*	
Children aged <5 y with fever only (n=80)	28 (35%)
Children aged <5 y with cough/fast breathing only (n=15)	2 (13%)
Children aged <5 y with diarrhea only (n=28)	12 (43%)
Children aged <5 y with multiple complaints (n=41)	22 (54%)
Children aged <5 y with other complaints (n=65)*	58 (89%)
All children aged <5 y with danger signs (n=229)	122 (53%)
*Children aged <5 y receiving appropriate prereferral management of danger signs disaggregated by age categories*	
Children aged 2–11 mo receiving appropriate care (n=178)	12 (21%)
Children aged 12–35 mo receiving appropriate care (n=178)	31(54%)
Children aged 36–60 mo receiving appropriate care (n=178)	14 (25%)
*Children aged <5 y receiving appropriate referral management of danger signs disaggregated by age categories*	
Children aged 2–11 mo receiving appropriate care (n=173)	38 (22%)
Children aged 12–35 mo receiving appropriate care (n=173)	79(46%)
Children aged 36–60 mo receiving appropriate care (n=173)	56 (32%)

*These patients were not eligible for prereferral treatment, which perhaps explains the higher rate of prereferral appropriateness.

Children in the age category of 12–35 mo comprised the highest number of cases of prereferral treatment (54%) and referral (46%), respectively. Children in the age category of 2–11 mo were less prereferred (21%) and therefore referred (22%) to care by CHWs (Table 2).

## Discussion

Danger signs pose a threat to the survival of children and can result in death if left unattended. The study findings show that the majority of patients with danger signs were managed appropriately and referred to facility-based care. Children aged <5 y were diagnosed and provided with prereferral treatment (where necessary); danger signs were identified and cases were referred to the nearest health facility in the catchment area of each CHW.^17^ Studies conducted in Uganda and other African countries are in agreement concerning the efforts of CHWs in providing proper care, offering prereferral treatment and overall disease management, especially for fever, cough and diarrhea, in accordance with the iCCM protocols.^21,22^

Fever was the most diagnosed danger sign among children who presented to a CHW in the study setting. Literature from Uganda and Congo has documented convulsions as a common danger sign. Cases of convulsion as a result of high fever among children aged <5 y in the region were numerous.^21,22^ In many parts of Uganda, fever which later manifests as malaria is an endemic condition; however, there are prevention and treatment programs in place for malaria.^23,24^

In the current study, fast breathing was less well managed, with fewer referred patients (40%) compared with fever and diarrhea. It is possible that CHWs offered amoxicillin medication to children aged <5 y after measuring the breath per min of the child and observed no danger signs in the chests of children with a cough in drawing in breath. This project emphasizes the importance of careful training and refresher training each quarter to ensure quality output. Needless to say, the use of time has been problematic for CHWs and some other health professionals in diagnosing chest difficulties in drawing in breath.^25,26^ This could one of the reasons why tests related to fast breathing and pneumonia are rare, despite this being one of the leading causes of mortality and morbidity among children aged <5 y in sub-Saharan Africa.^27^

CHWs are trained to manage diarrhea among children aged <5 y. The prevalence of diarrhea in this study was high. Many rural and urban settings of Uganda experience hygiene and sanitation challenges, a situation which contributes to rising cases of diarrhea.^28–30^ Studies have shown that diarrhea is a major concern in sub-Saharan Africa and Uganda in particular.^31,32^

There was a proportion of children who were not given prereferral treatment or referred at all in this study. CHWs might mistakenly have omitted to tick the fields of prereferral or referral in the CHW registers. Also, the possibility of human error cannot be ruled out. Because of the voluntary role of CHWs, occasionally they can be exhausted and/or can forget to do what is necessary. A study conducted in Ethiopia also established that, occasionally, CHWs prefer to treat their referred patients.^33^ Sometimes CHWs hardly recognize danger signs, and in some instances danger signs tend to resolve on their own, for example, vomiting if caused by a viral infection.^34^ Programmatically, through quarterly refresher training and support supervision of the CHWs, such challenges are identified and addressed.

The current study identified that the highest numbers of children who received prereferral treatment and were subsequently referred to the nearest health facility were aged 2–12 and 12–35 mo. A study in West Africa differs from this finding, reporting that children in the same age group had similar protection levels for malaria, especially in high transmission settings.^35^ In general, in this study setting, breastfeeding children and children aged ≤1 y are under parental care and are mostly carried on the back or in the arms of the caregiver.^36^ Diseases can be transmitted from the caregiver to the child. Children aged 12–35 mo are explorative once they start crawling, walking and playing with other children. This exposes them to fever, diarrhea and pneumonia in settings where such diseases are already prevalent.

### Limitations

Records from CHWs might be subject to information bias; however, that is expected for CHWs following quality assurance/refresher sessions during program implementation. Information recorded to a good level by CHWs reflects their actual management of children with danger signs. CHW registers do not give details on specific danger signs and are limited by their design. This makes analysis of specific danger signs (e.g. convulsions and chest difficulties in drawing in breath) incomplete.

### Conclusions

CHWs play a key role in early symptomatic detection, prereferral treatment and early referral of children aged <5 y. If left untreated, danger signs in children aged <5 y can result in death. The majority of children were managed appropriately by the CHWs as per the iCCM protocol. There is a need to ensure that all children aged <5 y are referred as required. This calls for identification and mitigation of barriers to the proper management of children with fast breathing presenting to CHWs. More studies need to focus on why children aged 2–11 and 12–35 mo were the most affected by danger signs. Improving the health outcomes of children with danger signs can contribute towards achieving WHO Sustainable Development Goal 3.2.

## Data Availability

All data supporting the study findings are contained in the paper. There are no restrictions to the data sources, however full details to the data may be accessed on reasonable request from the corresponding author.
